# Supplementary material for: Using machine learning analysis to interpret the relationship between music emotion and lyric features
Source: PeerJ Comput Sci. 2021 Nov 15;7:e785. doi: 10.7717/peerj-cs.785 (PMC8627224; doi:10.7717/peerj-cs.785)
Supplement: Supplemental Information 1 [file peerj-cs-07-785-s001.docx]

**Supplemental Materials**

**Contents**

[**Table S1**. Introduction of SC-LIWC based lyric features in this study. 2](#_Toc83837009)

[**Table S2**. Correlation results between lyric features and perceived emotions 5](#_Toc83837010)

[**Table S3.** Correlation between audio features and *WordCount.* 7](#_Toc83837011)

[**Figure S1**. The relationship between music duration and arousal. 8](#_Toc83837012)

## **Table S1**. Introduction of SC-LIWC based lyric features in this study.

| **Id** | **Feature name** | **Feature description** |
| --- | --- | --- |
| 1 | *Funct* | Proportion of function words, including negative words, quantifier, number, and tense calibration words. |
| 2 | *Pronoun* | Proportion of pronoun. |
| 3 | *PPron* | Proportion of personal pronouns. |
| 4 | *I* | Proportion of first-person singular pronouns. |
| 5 | *We* | Proportion of first-person plural pronouns. |
| 6 | *You* | Proportion of second-person pronouns. |
| 7 | *SheHe* | Proportion of third-person singular pronouns. |
| 8 | *They* | Proportion of third-person plural pronouns. |
| 9 | *iPron* | Proportion of impersonal pronouns. |
| 10 | *Article* | Proportion of articles. |
| 11 | *Verb* | Proportion of verbs. |
| 12 | *AuxVerb* | Proportion of auxiliary verbs. |
| 13 | *enPast* | Proportion of past tense words (English). |
| 14 | *enPresent* | Proportion of present tense words (English). |
| 15 | *enFuture* | Proportion of future tense words (English). |
| 16 | *Adverb* | Proportion of adverbs. |
| 17 | *Preps* | Proportion of preposition. |
| 18 | *Conj* | Proportion of conjunction. |
| 19 | *Negate* | Proportion of negative words. |
| 20 | *Quant* | Proportion of function quantifier. |
| 21 | *Number* | Proportion of number. |
| 22 | *Swear* | Percentage of swear words. |
| 23 | *YouPL* | Proportion of second-person plural pronouns. |
| 24 | *PrepEnd* | Proportion of postposition words. |
| 25 | *SpecArt* | Proportion of specific words. |
| 26 | *QuanUnit* | Proportion of quantity unit words. |
| 27 | *Interjunction* | Proportion of interjunction. |
| 28 | *MultiFun* | Proportion of multi-function words |
| 29 | *TenseM* | Proportion of tense markers. |
| 30 | *PastM* | Proportion of past tense markers. |
| 31 | *PresentM* | Proportion of present tense markers. |
| 32 | *FutureM* | Proportion of future tense markers. |
| 33 | *ProgM* | Proportion of words for continuation. |
| 34 | *Social* | Proportion of words related to social relations, including family, friend, and humans. |
| 35 | *Family* | Proportion of words related to family. |
| 36 | *Friend* | Proportion of words related to friend. |
| 37 | *Humans* | Proportion of words related to humans. |

**Table S1** (continued)

| **Id** | **Feature name** | **Feature description** |
| --- | --- | --- |
| 38 | *Affect* | Proportion of words related to affect, including positive and negative emotion words. |
| 39 | *PosEmo* | Proportion of positive emotion words. |
| 40 | *NegEmo* | Proportion of negative emotion words. |
| 41 | *Anx* | Proportion of words related to anxiety. |
| 42 | *Anger* | Proportion of words related to anger. |
| 43 | *Sad* | Proportion of words related to sadness. |
| 44 | *CogMech* | Proportion of words related to cognition, consisting of words related to insight, cause, discrepancy, tentativeness, certainty, inhibition, inclusiveness, and exclusiveness. |
| 45 | *Insight* | Proportion of words related to insight. |
| 46 | *Cause* | Proportion of words related to cause. |
| 47 | *Discrep* | Proportion of words related to discrepancy. |
| 48 | *Tentat* | Proportion of words related to tentativeness. |
| 49 | *Certain* | Proportion of words related to certainty. |
| 50 | *Inhibition* | Proportion of words related to inhibition. |
| 51 | *Inclusive* | Proportion of inclusive words. |
| 52 | *Exclusive* | Proportion of exclusive words. |
| 53 | *Percept* | Proportion of words related to perception, consisting of words related to vision, hearing, and feeling. |
| 54 | *See* | Proportion of words related to vision. |
| 55 | *Hear* | Proportion of words related to hearing. |
| 56 | *Feel* | Proportion of words related to feeling. |
| 57 | *Bio* | Proportion of words related to biology, consisting of words related to body, health, sexual, and ingest. |
| 58 | *Body* | Proportion of words related to body. |
| 59 | *Health* | Proportion of words related to health. |
| 60 | *Sexual* | Proportion of words related to sexual. |
| 61 | *Ingest* | Proportion of words related to ingest. |
| 62 | *Relative* | Proportion of relative words, consisting of words related to motion, space, and time. |
| 63 | *Motion* | Proportion of words related to motion. |
| 64 | *Space* | Proportion of words related to space. |
| 65 | *Time* | Proportion of words related to time. |
| 66 | *Work* | Proportion of words related to work. |
| 67 | *Achieve* | Proportion of words related to achievement. |
| 68 | *Leisure* | Proportion of words related to leisure. |
| 69 | *Home* | Proportion of words related to home. |
| 70 | *Money* | Proportion of words related to money. |
| 71 | *Religion* | Proportion of words related to religion. |
| 72 | *Death* | Proportion of words related to death. |

**Table S1** (continued)

| **Id** | **Feature name** | **Feature description** |
| --- | --- | --- |
| 73 | *Assent* | Proportion of words related to assent. |
| 74 | *Nonfl* | Proportion of pause superfluous words. |
| 75 | *Filler* | Proportion of superfluous fillers. |
| 76 | *Psychology* | Proportion of words related to psychology. |
| 77 | *Love* | Proportion of words related to love. |
| 78 | *tPast* | Proportion of words related to the past. |
| 79 | *tNow* | Proportion of words related to the present. |
| 80 | *tFuture* | Proportion of words related to the future. |
| 81 | *Period* | Proportion of period. |
| 82 | *Comma* | Proportion of comma. |
| 83 | *Colon* | Proportion of colon. |
| 84 | *SemiC* | Proportion of semicolon. |
| 85 | *QMark* | Proportion of question mark. |
| 86 | *Exclam* | Proportion of exclamation mark. |
| 87 | *Dash* | Proportion of dash. |
| 88 | *Quote* | Proportion of quotation marks. |
| 89 | *Apostrophe* | Proportion of abbreviation. |
| 90 | *Parenth* | Proportion of brackets. |
| 91 | *OtherP* | Proportion of other punctuation. |
| 92 | *WordCount* | The total number of words. |
| 93 | *WordPerSentence* | Average number of words per sentence. |
| 94 | *RateDicCover* | Coverage of SC-LIWC. |
| 95 | *RateNumeral* | Proportion of numbers. |
| 96 | *RateSixLtrWord* | The ratio of Chinese word length greater than or equal to 6. |
| 97 | *RateFourCharWord* | The ratio of Chinese word length greater than or equal to 4. |
| 98 | *RateLatinWord* | The ratio of Latin words. |

## **Table S2**. Correlation results between lyric features and perceived emotions

| **Lyric features** | **Arousal** | **Valence** | **Lyric features** | **Arousal** | **Valence** |
| --- | --- | --- | --- | --- | --- |
| *NegEmo* | -.073** | -.364** | *Friend* | 0.01 | .082** |
| *Sad* | -.055** | -.299** | *Religion* | 0.007 | .081** |
| *CogMech* | -.098** | -.271** | *OtherP* | 0.027 | .079** |
| *Tentat* | -.087** | -.243** | *Health* | 0.034 | -.077** |
| *Adverb* | -.069** | -.239** | *PrepEnd* | -0.019 | .075** |
| *PastM* | -.121** | -.223** | *Anx* | -0.018 | -.075** |
| *TenseM* | -.100** | -.214** | *Social* | -0.026 | -.074** |
| *tPast* | -.124** | -.209** | *You* | -.070** | -.073** |
| *Insight* | -.122** | -.202** | *Comma* | -.069** | .072** |
| *Exclusive* | -.056** | -.202** | *Exclam* | 0.035 | .067** |
| *See* | 0.034 | .194** | *Body* | .053** | .066** |
| *ProgM* | -.063** | -.186** | *FutureM* | -0.029 | -.065** |
| *Funct* | -.055** | -.182** | *Filler* | .087** | .059** |
| *AuxVerb* | -.052* | -.172** | *Parenth* | 0.029 | .059** |
| *Discrep* | -.064** | -.165** | *SheHe* | 0.003 | -.054** |
| *Verb* | -0.039 | -.165** | *Interjunction* | 0.033 | .053* |
| *Space* | .049* | .164** | *Achieve* | .111** | .051* |
| *Percept* | 0.012 | .158** | *Dash* | -.047* | -.050* |
| *Leisure* | .071** | .156** | *Relative* | -0.007 | .050* |
| *Time* | -.115** | -.152** | *RateSixLtrWord* | 0.007 | .048* |
| *RateDicCover* | -.047* | -.152** | *tNow* | -0.023 | -.046* |
| *RateLatinWord* | .183** | .148** | *They* | 0.037 | .044* |
| *PosEmo* | .109** | .145** | *Love* | -0.018 | -.044* |
| *iPron* | -.043* | -.138** | *Nonfl* | -0.003 | .044* |
| *Cause* | -0.008 | -.138** | *Period* | -0.036 | .041* |
| *Conj* | -.054** | -.136** | *Humans* | 0.009 | -0.039 |
| *Pronoun* | -0.036 | -.136** | *RateNumeral* | .072** | 0.035 |
| *Inhibition* | -.089** | -.134** | *WordPerSentence* | .179** | -0.034 |
| *Psychology* | -.061** | -.133** | *YouPL* | 0.008 | 0.031 |
| *Affect* | 0.021 | -.132** | *Money* | .063** | 0.03 |
| *Family* | .098** | .131** | *Preps* | -0.02 | -0.028 |
| *Motion* | .082** | .118** | *Colon* | 0.007 | 0.027 |
| *Anger* | .055** | -.115** | *Feel* | 0 | 0.027 |
| *Negate* | -.054** | -.108** | *QMark* | -.051* | 0.025 |
| *We* | .044* | .108** | *PresentM* | -0.005 | 0.025 |
| *Certain* | -0.011 | -.107** | *QuanUnit* | -0.013 | 0.023 |
| *Quant* | 0.034 | .101** | *MultiFun* | 0.034 | 0.021 |
| *RateFourCharWord* | 0.039 | .099** | *SemiC* | -0.021 | 0.021 |
| *Sexual* | 0.018 | -.096** | *Apostrophe* | -0.023 | 0.02 |

**Correlation is significant at the 0.01 level (2-tailed).

*Correlation is significant at the 0.05 level (2-tailed).

**Table S2** (continued)

| **Lyric features** | **Arousal** | **Valence** | **Lyric features** | **Arousal** | **Valence** |
| --- | --- | --- | --- | --- | --- |
| *Death* | .054** | -.094** | *Number* | -0.006 | 0.02 |
| *Work* | .072** | .092** | *Quote* | -0.026 | -0.018 |
| *I* | 0.021 | -.092** | *Hear* | 0 | 0.017 |
| *Inclusive* | 0.012 | .092** | *Swear* | 0.017 | -0.015 |
| *Ingest* | .052* | .090** | *Bio* | .070** | -0.014 |
| *PPron* | -0.019 | -.089** | *SpecArt* | -0.003 | -0.011 |
| *Assent* | 0.007 | -.083** | *tFuture* | 0.017 | 0.01 |
| *Home* | 0.011 | .082** | *WordCount* | .206** | 0.006 |

**Correlation is significant at the 0.01 level (2-tailed).

*Correlation is significant at the 0.05 level (2-tailed).

## **Table S3.** Correlation between audio features and *WordCount.*

|  | 1 | 2 | 3 | 4 | 5 | 6 | 7 | 8 | 9 |
| --- | --- | --- | --- | --- | --- | --- | --- | --- | --- |
| 1. *WordCount* | 1 |  |  |  |  |  |  |  |  |
| 2. MFCCs 1 | -.284** | 1 |  |  |  |  |  |  |  |
| 3. chromagram 1 | .281** | -.600** | 1 |  |  |  |  |  |  |
| 4. spectral contrast 1 | -.216** | .637** | -.683** | 1 |  |  |  |  |  |
| 5. MFCCs 2 | -.168** | -0.032 | .166** | -.159** | 1 |  |  |  |  |
| 6. spectral centroid 1 | -.162** | .343** | -.356** | .254** | -.121** | 1 |  |  |  |
| 7. spectral flatness 1 | .157** | -.532** | .676** | -.669** | .199** | .084** | 1 |  |  |
| 8. spectral contrast 9 | .102** | -0.017 | 0.026 | 0.022 | -.303** | -0.01 | -0.011 | 1 |  |
| 9. spectral flatness 29 | .096** | -0.018 | -0.002 | 0.008 | -.088** | -0.022 | -0.034 | 0.01 | 1 |

This table only shows eight audio features most correlated with *WordCount.*

**Correlation is significant at the 0.01 level (2-tailed).

*Correlation is significant at the 0.05 level (2-tailed).





## **Figure S1**. The relationship between music duration and arousal.

Error bars indicate standard errors.
